# Lithotrity and Lithotomy

**Published:** 1851-12

**Authors:** 


### THE WESTERN JOURNAL.

THIRD SERIES.—VOL. VIII.—NO. VI.

LOUISVILLE, DECEMBER 1, 1851.

### LITHOTRITY AND LITHOTOMY.

The readers of this *Journal* will remember that upon a former occasion, an extraordinary case of lithotrity, reported by Prof. Dugas, editor of the *Southern Medical and Surgical Journal*, published at Augusta, Georgia, was republished in this *Journal*, with some comments, by the junior editor. The remarks were conceived in no ill spirit, they were such as the conductor of a scientific journal had a perfect right to make, and such as legitimate criticism, fully justified. The lithotritist who reports wonderful and singular cases, as occurring within the province of his specialty, must expect that he who has great faith in lithotomy, and not a very abundant belief in lithotrity, will demand a rigid exactness in the publication of cases that are designed to sustain what, such a person as the writer describes, believes to be an error. The commentary alluded to, though appended to a full republication of the report made by Prof. Dugas, was seized upon in the absence of the Professor, for as vindictive and malevolent a rejoinder, by a colleague of Prof. Dugas, as ever adorned the brilliant columns of a partisan newspaper. Nothing could tempt the writer of the remarks upon Prof. Dugas' extraordinary case, to be the author of such a piece of unadulterated railing and abuse, as the rejoinder alluded to. It complaicently purported to be a defense of Prof. Dugas' case, when it was nothing more nor less, than one continuous stream of coarse invective. It was not until after the publication of the original commentary upon Professor Dugas' case, that the writer knew of his absence, and on account of that absence, the Professor's volunteer champion was permitted to rail to his heart's content.

Prof. Dugas seems to regard the production of his champion very much as others did—as a failure, for since his return, he has taken the matter in hand, and, in the November number of the *Southern Medical and Surgical Journal*, has reopened the question. Whether he has bettered the issue remains to be seen.

In the multiplicity of extraordinary cases prepared for medical journals, it has rarely been the lot of the writer to meet one bordering more on the marvelous than that reported by Prof. Dugas. About the time of the advent of that remarkable case, a distinguished medical gentleman, greatly distinguished as a writer for medical periodicals, published a wonderful case of the evacuation of the contents of an ovarian cyst, by the catheterism of the Fallopian tubes. That gentleman does not seem to have had his equanimity disturbed by the expression of doubts as to the truth of his report. The field for doubt was infinitely wider than the field for faith, and the reporter of that case seemed to consider that his medical brethren had a right to exercise their unbelief. He has not claimed, anywhere publicly, any peculiar grace for not making a personal assault upon those who were dubious of his claims. But Professor Dugas seems to consider it a very meritorious badge of his editorial dignity, that he does not enter into personalities towards his medical critic! Amazing sacrifice! How transcendently worthy of honor and regard is that spirit of science, that soars in its lofty eyrie, and does not descend to sweep the vales of humble life, with its terrible talons! How grateful should the escaped victim be! Scinentific lucubrations rather than personal invective!

The main gist of Professor Dugas' defense is upon a point on which there seems to be an antagonism between the volunteer champion and the Professor. One of the greatest difficulties expressed in the original commentary was in respect to the extraordinary dilatation of the ureters, and the non-synchronism of the urethra in that dilatation. This vexatious point seemed to be prominently set forth in the report of Professor Dugas, and was evidently a *pous asinorum* to the fiery defender of the case of lithotrity. Instead of denying that Professor Dugas had made such a representation as that ascribed to him, the champion, filled with the most generous impulses of a very ardent faith, was rather disinclined to the labor of explanation, and very coolly cut the Gordian Knot, by declaring he could explain the whole affair, if he had *patience!* This seemed to be a great aggravation of the whole matter. Here was confessedly one of the greatest of pathological riddles that had ever loomed upon vision, and an œdipus walked the earth, capable of solving the riddle of the sphynx, but he would not do it, because he was fretful!—The case seemed a very hard one, but there was no help. The oracle declared it had not the patience to explain, and dullness had to grope on in darkness.

It is unnecessary to dwell on this subject—those who feel any kind of interest in the matter can easily turn to the number of this Journal which contained Prof. Dugas' report of his case of lithotrity, together with our comment on it, and can see whether any injustice was done to the Professor. His report plainly expressed by implication what the comment represented. There was a claim set up of an extraordinary dilation of the ureters, accompanied with the statement that the subject of dilatation had long been the subject of phymosis, from which phymosis he had been relieved by an operation. The writer has never yet seen any one who did not draw the same inference from reading Professor Dugas' report that he did, nor has he found any one to draw any other inference, even with the aid of the Professor's new light on the subject.

Nor has Professor Dugas succeeded any better, in his new effort to prove the dilatation of the ureters. He assumed that the ureters were greatly dilated, because the calculus which he found in the bladder had passed through them. But it seems to be all assumption. There is no proof that the ureters were dilated in any degree, for it is very far from being certain that the calculus was ever in the pelvis of the kidneys.—The investigations of a very high authority on such subjects, quoted in the original commentary, seemed to be conclusive on the question, and have not been impugned, either by Professor Dugas' volunteer champion, nor by the Professor in his appendix to the work of his brother Professor.

The facile destruction of an oxalate of lime calculus, claimed by Professor Dugas, transcending as it does, all former feats of lithotrity, even transcending the legerdemain reports of Heurteloup himself, was well calculated to make readers pause. No one is compelled to yield his assent to the truth of all the opinions and inferences of a medical writer, no matter how respectable he may be. The most respectable of men, have been in error, even in their most pains-taking efforts. The

Professor attempts to bolster himself by a certificate, but certificates are not cures, nor can all the certificates in Georgia shake the well expressed declaration of Malgaigne—one month is entirely too short a period in which to claim a cure of a calculous disease, when the philosophic and experienced Malgaigne says, twelve months should elapse between the operation, and the claim of a cure. Upon that point the critic holds himself invulnerable, if the great experience of Malgaigne is any authority against the opinions of a mere neophyte in lithotrity. The investigations of Dr. Aldridge, and the experience of Malgaigne are antagonistic to the views of Professor Dugas, on two vital points of his case, and until those two authorities are met and refuted in some way, even the indulgence of personalities on the part of Professor Dugas, towards his critic, can do the cause of the Professor no kind of service. Let Malgaigne and Aldridge be successfully refuted, and Prof. Dugas' critic will yield the case. But while they stand, the critic's case will prove impregnable. B.

P. S.—Long after the foregoing remarks were in type, and this form was, in printer's phrase, made up, a note from Professor Dugas was placed in the hands of the writer of these remarks, requesting the republication of Prof. D's. rejoinder to the criticism that was made on his case. It is due to Professor Dugas and to his critic to make this explanation, and it is obvious to the Professor why no allusion to his polite note was made in the reply to which this is a postscript. The request is under advisement, because it assumes that some injustice was done to Professor Dugas in the original commentary. Wherein that injustice consists is not very palpable to us. If we had assailed Professor Dugas's report, without permitting our readers to see that report, he would have had just cause of complaint. If we had garbled his report, and republished such portions only as suited our notions for attack, we should admit the force of the Professor's complaint. But we neither suppressed the Professor's report, nor garbled it, but published it in full, at the head of our commentary. Our readers are competent to decide whether our commentary was justified by Professor Dugas's report, for they had the report with the commentary. The author of the commentary was bitterly and personally assailed in Professor Dugas's Journal, by one of the Professor's colleagues, and has never had "justice" extended to him, by any allusion even to the fact, that a reply was made to the invective of the Professor's friend.

The editor of the *New York Medical Gazette* seems disposed to do Professor Dugas more than justice. He shouts *Io peans* over each allusion of Professor Dugas's Journal to this matter, and with a remarkably cool sapiency announces, that the review of Professor Dugas's report, in this Journal, was made anonymously!!

